# Roles of *ARID1A* variations in colorectal cancer: a collaborative review

**DOI:** 10.1186/s10020-022-00469-6

**Published:** 2022-04-14

**Authors:** Shankun Zhao, Weizhou Wu, Zufu Jiang, Fuqin Tang, Lingzhi Ding, Weifang Xu, Libin Ruan

**Affiliations:** 1 Department of Urology, Taizhou Central Hospital (Taizhou University Hospital), Taizhou, 318000 Zhejiang China; 2 Department of Urology, Maoming People’s Hospital, Maoming, 525000 Guangdong China; 3 Department of General Surgery, Taizhou Central Hospital (Taizhou University Hospital), Taizhou, 318000 China; 4 Nursing Department, Taizhou Central Hospital (Taizhou University Hospital), Taizhou, China; 5 Department of Orthopedics, Taizhou Central Hospital (Taizhou University Hospital), Taizhou, 318000 Zhejiang China

**Keywords:** *ARID1A* variations, Colorectal cancer (CRC), Biomarker, Prognosis, Pathogenesis

## Abstract

Colorectal cancer (CRC), a common malignancy, is one of the leading cause of cancer death in adults. AT-rich interaction domain 1A (*ARID1A*), a critical portion of the SWItch/sucrose non-fermentation (SWI/SNF) chromatin remodeling complexes, shows one of the most frequent mutant genes across different human cancer types. Deleterious variations of *ARID1A* has been recognized to be correlated the tumorigenesis and the poor prognosis of CRC. Here, we summarize recent advances in the clinical implications and molecular pathogenesis of *ARID1A* variations in CRC. According to independent data of 23 included studies, *ARID1A* is mutated in 3.6–66.7%. Consistently, all of the 23 relevant studies report that *ARID1A* functions as a specific tumor suppressor in CRC. Clinically, *ARID1A* variation status serves as a biomarker for survival prognosis and various therapies for CRC. Mechanistically, the pathophysiologic impacts of *ARID1A* variations on CRC may be associated with the co-occurrence variations of other genes (i.e., TP53, KRAS, APC, FBXW7, and PIK3CA) and the regulation of several signaling pathways being affected (i.e., WNT signaling, Akt signaling, and MEK/ERK pathway), leading to cell cycle arrest, chromatin remodeling, chromosome organization, and DNA hypermethylation of the cancer cells. The present review highlights *ARID1A* serving as a potent tumor suppressor and an important prognostic factor in CRC. *ARID1A* variations hint towards a promising tool for diagnostic tumor profiling and individualized therapeutic targets for CRC in the future.

## Introduction

Colorectal cancer (CRC) is one of the top-common malignancies worldwide, nearly 1,850,000 incidences, and is the second leading cause of cancer deaths, with an approximate 881,000 fatalities (9.2% of all fatal cancer cases) in the world yearly (Bray et al. 2018). CRC with locoregional lymph node diffuse has a 5-year overall survival (OS) of 70% while disuse to distant organs carries a substantially worse prognosis with a 5-year OS of 12% (Siegel et al. 2018). Metastasis to the liver is the most common site of distant spread (Fong et al. 1997) while the peritoneal surface is the second most common site of metastasis, involving roughly 10% of patients with CRC at the very beginning of the presentation and the sole site of recurrence in as much as 25% of patients with CRC (Dawson et al. 1983; Russell et al. 1983). Peritoneal metastasis (PM) is associated with a poor prognosis, and the survival period for systemic chemotherapy alone is 5–7 months (Chu et al. 1989; Koppe et al. 2006). Compared to other site transfers, PM is associated with a greatly shorter progression-free survival (PFS) and OS (Franko et al. 2012). The molecular underlying mechanisms of CRC is driven by the continuous acquisition of epigenetic and genetic abnormalities, which is related to the repression of the tumor suppressor and the activation of pro-oncogenic factors (Lao and Grady 2011). The low effectiveness of conventional therapeutic interventions to prolong life span in CRC patients needs new and effective targeted therapies.

The heterogeneity of CRC tumor aggressiveness and prognosis might be prompted by differences in genetic variation. According to some reports, the gene encoding the SWItch/sucrose non-fermentation (SWI/SNF) chromatin remodeling complex is one of the most common mutant genes in a variety of malignant tumors. SWI/SNF chromatin remodeling complex play role in the transcription and DNA reproduction and repair (Wilson and Roberts 2011). Among the family of the SWI/SNF genes, AT-rich interaction domain 1A (*ARID1A*) is a common-mutated gene in human cancers, which contributes to the binding of protein and DNA (Kadoch and Crabtree 2013; Wang et al. 2004). ARID1A, a gene located on chromosome 1p36.11, is a core component of the mammalian SWI/SNF complex (Megaridis et al. 2018). ARID1A encodes a protein with nuclear/cytosolic localization. Nuclear ARID1A is speedily degraded by the nuclear ubiquitin–proteasome system unstable due to the nuclear ARID1A is unstable (Mao and Shih 2013). In-frame deletions disrupting the nuclear export signal cause a declination of ARID1A expression, due to the nuclear retention of the protein and its subsequent degradation (Mao and Shih 2013; Guan et al. 2012). ARID1A exhibits its biological function by interacting with DNA and recruiting associated transcriptional co-activators, while ARID1A variation commonly cause the dysregulation of BAF complex-mediated chromatin remodeling (Chandler et al. 2013). ARID1A contains an ARID domain, which interacts with DNA in a sequence-nonspecific manner modulating cellular processes (e.g., proliferation and differentiation) (De and Dey 2019). Thus, ARID1A has been found to be contributed to the tumorigenesis of multiple cancers.

*ARID1A* has lately been recognized as a crucial tumor suppressor gene in diverse cancer types. Ovarian cancer, stomach cancer, and pancreatic cancer have the highest mutation (or variation) frequency (29–57%), while CRC (13%), liver cancer (10–17%), bladder cancer (13%), esophageal cancer (9%), breast cancer (3%) and childhood retinoblastoma (6%) have somewhat lower variation frequencies (Cornen et al. 2012; Dulak et al. 2013; Fujimoto et al. 2012; Gui et al. 2011; Guichard et al. 2012; Jones et al. 2012, 2010; Sausen et al. 2013; Shain et al. 2012; Wiegand et al. 2010). Also, Ogiwara et al. (2019) summarized that ARID1A is mutated in about 46% of ovarian clear cell carcinomas, 43% of uterine corpus endometrial carcinomas, 33% of gastric carcinomas, 30% of ovarian endometrioid carcinomas, 28% of bladder carcinomas, 27% of cholangiocarcinomas, 15% of pancreatic carcinomas, 12% of lung adenocarcinomas, and 10% of CRC. The frequency of ARID1A variations in ovarian clear cell carcinomas is up to 60% in the US, Canada, and Japan, indicating that ARID1A deficiency may be a potential biomarker for precision medicine of ovarian cancer (Takahashi et al. 2021). It was reported that ARID1A variations was observed in up to 40% of low-grade endometrioid carcinomas (Toumpeki et al. 2019). The reported ARID1A mutant prevalence in gastric cancer among different studies was 8–27% (Wang et al. 2021). Dugas et al. (2019) demonstrated that ARID1A variation was observed in 3.6% of the non-muscle-invasive bladder cancer and 10% of the muscle-invasive bladder cancer. Zhao et al. showed that the variation rate of ARID1A in cholangiocarcinomas ranged from 5% to 68.2% (Zhao et al. 2021). Though ARID1A may be not the most highly mutated gene in the aforementioned malignancies, it can synergize with other mutant genes to promote the pathogenesis and the development of cancers.

Most of the *ARID1A* variations are inactive condition that result in the loss of the protein expression of *ARID1A* (Kishida et al. 2019). In current years, mounting evidence revealed that *ARID1A* variation is related to the clinicopathologic characteristics of CRC (Wei et al. 2014; Ye et al. 2014). At present, in different clinical studies, the specific role of *ARID1A* on the prognosis and clinicopathological features of CRC is widely debated. According to published data, most studies indicate that *ARID1A* serves as an important tumor suppressor gene. For example, Lee et al. (2016) demonstrated that no connection was evident between *ARID1A* expression and 5-year OS. However, a recent study conducted by Jiang et al. (2020) showed that disease-free or PFS of patients with *ARID1A* variations [DFS/PFS, HR = 0.74 (0.64–0.91), *P* = 0.0026]. The OS of patients with *ARID1A* variations was significantly prolonged by 28 months, compared with 18 months in those with wild-type *ARID1A* [HR = 0.73 (0.61–0.93), *P* = 0.0092]. The role of *ARID1A* in CRC is currently uncertain. In this narrative review, we aim to overview all the current evidence that *ARID1A* variation or expression is associated with the development of CRC, and reveal the potential molecular mechanisms.

### Searching strategy

Four common data bases were searched to find the eligible studies prior to January 1, 2022. The searching strategy these databases was: ((((((((((((((ARID1A) OR (B120)) OR (BAF250)) OR (BAF250a)) OR (BM029)) OR (C1orf4)) OR (CSS2)) OR (ELD)) OR (MRD14)) OR (OSA1)) OR (P270)) OR (SMARCF1)) OR (hELD)) OR (hOSA1)) AND ((((((((((((((((((((“Colorectal Neoplasms”[Mesh]) OR (Colorectal Neoplasm)) OR (Neoplasm, Colorectal)) OR (Neoplasms, Colorectal)) OR (Colorectal Tumors)) OR (Colorectal Tumor)) OR (Tumor, Colorectal)) OR (Tumors, Colorectal)) OR (Colorectal Cancer)) OR (Cancer, Colorectal)) OR (Cancers, Colorectal)) OR (Colorectal Cancers)) OR (Colorectal Carcinoma)) OR (Carcinoma, Colorectal)) OR (Carcinomas, Colorectal)) OR (Colorectal Carcinomas)) OR (Colonic Neoplasm)) OR (Colon Cancer)) OR (Rectal Neoplasms)) OR (Rectum Cancer)). For identifying more eligible studies, we manually inspected the reference lists in the related articles. According to the data collection form, the following information in each study was extracted, including the first authors’ names, the publication year, study area, type of CRC, *ARID1A* variations in CRC, and some details of clinical and molecular aspects.

**Fig. 1:**
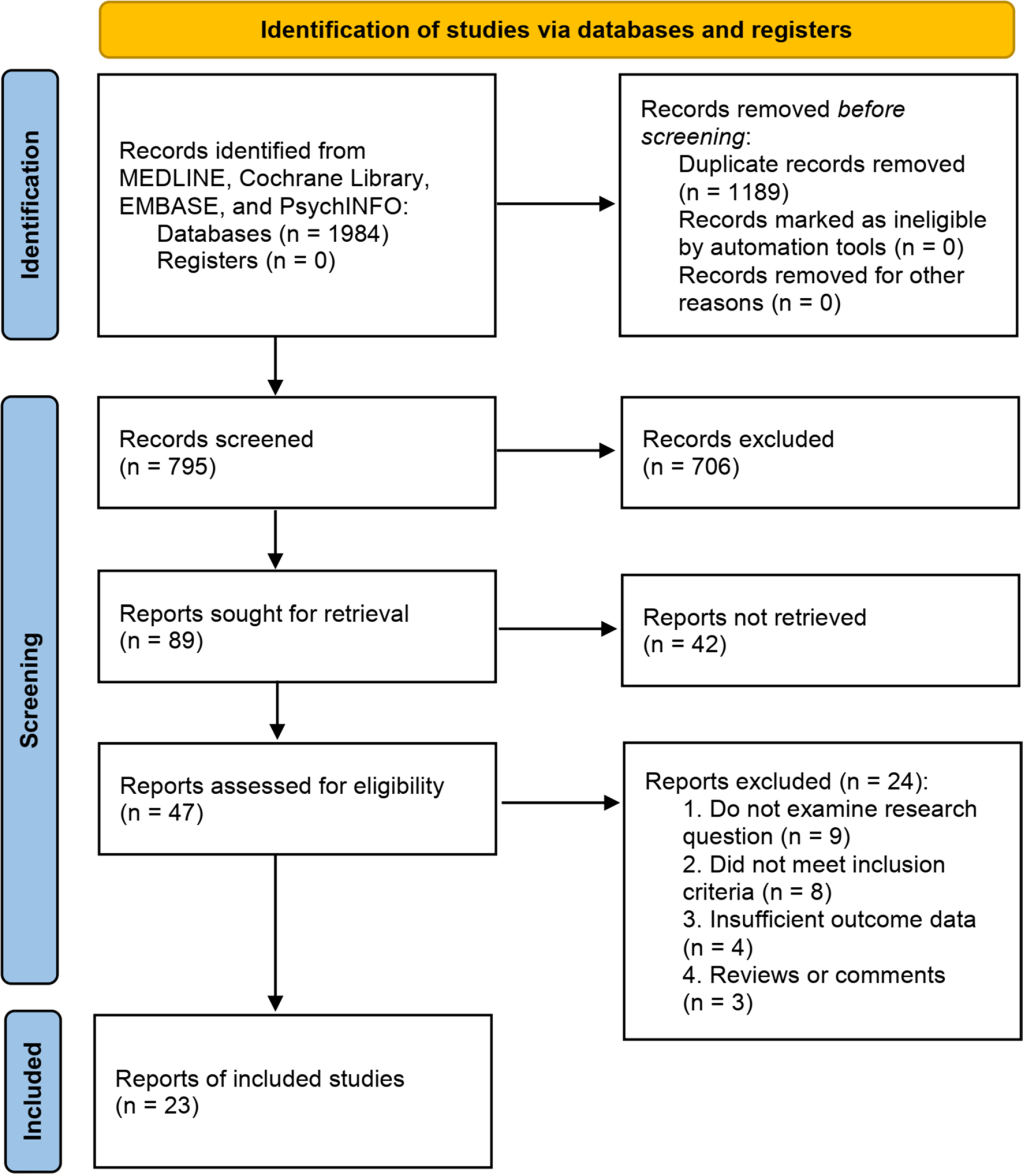
Flow chart of study selection

**Fig. 2:**
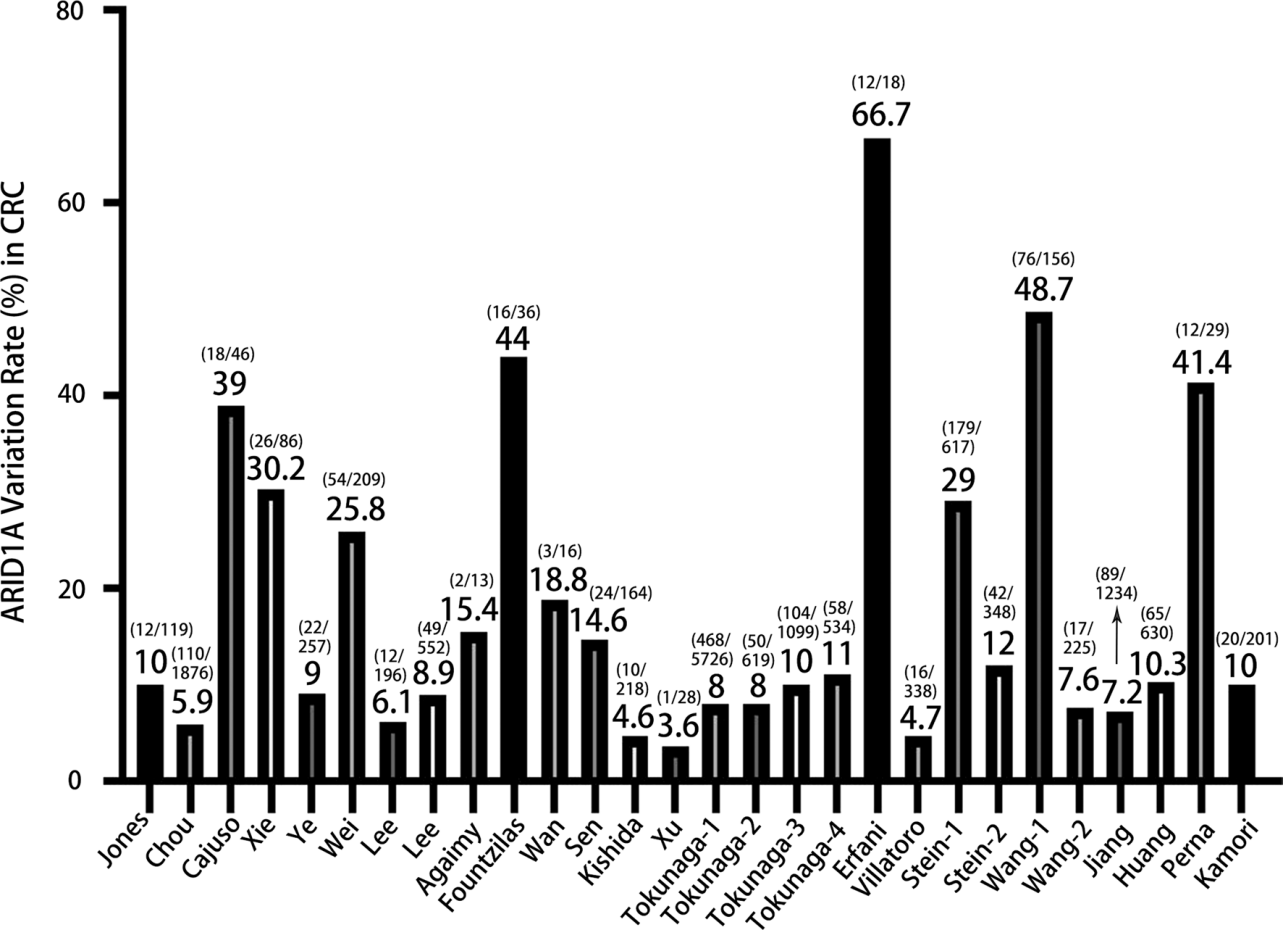
Variation rate of *ARID1A* in CRC among different studies

Figure 1 showed the search flowchart. Finally, 23 eligible studies (16, 23–44) with a total of 15,580 subjects were included. The characteristics of the 23 eligible studies were listed in Table 1. According to the available information from the 23 included studies, ARID1A variation was defined as the loss or low expression levels of ARID1A. Therefore, though “ARID1A mutation” could be found several previous related studies, ARID1A is in fact variant rather than mutated. Besides, according to the ACMG guidelines for nomenclature of the genomic variations, it is recommended to use the term “variation” instead of “mutation” (Richards et al. 2015). As shown in Fig. 2, the frequency of *ARID1A* variations among the 23 studies ranged from 3.6% to 66.7%. The mutant type is defined as the loss or low of *ARID1A* protein expression. Jones et al. (2012), suggested that *ARID1A* has a tumor suppressor function in the pathogenesis of CRC, and reported that *ARID1A* expression reduction and/or somatic variations are associated with the progression of CRC. In Cajuso et al.’s study (Cajuso et al. 2014), they used exome sequencing data to investigate the variation frequency of all genes containing the drought domain in 25 cases of microsatellite unstable (MSI) CRC. The authors identified 47 different somatic variations, including 18 frameshift (38%, c.5548delG), 3 nonsense (6%), 18 missense (38%), 1 splice site (2%), and 7 silent mutation (15%).

**Table 1:** The characteristics of the 23 eligible studies

Author and country	Publication year	Type of CRC	*ARID1A* variation (%)	*ARID1A* expression	Roles of *ARID1A*	Clinicopathologic features or biological effects of *ARID1A*	Antibodies of *ARID1A*	References
Jones, USA	2012	CRC	12/119, 10%	Downregulated	Suppressor	*ARID1A* inactivation promoted CRC development	NA	Jones et al. (2012)
Chou, Australia	2014	CRC	110/1876, 5.9%;	Loss of expression	Suppressor	No significant relationship between loss expression of *ARID1A* and the OS of CRC; Loss expression of *ARID1A* was associated with multiple clinical features, BRAFV600E variation and loss of mismatch repair protein expression (all P < 0.01)	Sigma 1: 100	Chou et al. (2014)
Cajuso, Finland	2014	CRC	18/46, 39%	Downregulated	Suppressor	Exome sequencing showed that *ARID1A* play an important role in microsatellite-unstable CRC via DNA binding activity and transcription coactivator activity	Santa Clara	Cajuso et al. (2014)
Xie, China	2014	CRC	26/86, 30.2%	Loss of expression	Suppressor	Loss of *ARID1A* significantly associated with poor differentiation of CRC (P = 0.0009)	Rabbit antibodies Sigma 1:500	Xie et al. (2014)
Ye, USA	2014	CRC	22/257, 9%	Loss of expression	Suppressor	*ARID1A* loss was significantly associated with various clinicopathological features of CRC (all P < 0.05), and with a trend toward a worse OS (P > 0.05)	polyclonal antibody Sigma- 1:100	Ye et al. (2014)
Wei, China	2014	CRC	54/209, 25.8%	Loss of expression	Suppressor	*ARID1A* loss was correlated to late TNM stage, distant metastasis, and poor pathological classification (all P < 0.05)	Santa Cruz Biotechnology	Wei et al. (2014)
Lee, Korea	2015	CRC	12/196, 6.1%	Loss of expression	Suppressor	Loss of *ARID1A* expression was significantly correlated with negative lymphatic invasion (P = 0.003) in CRC, and with expanding tumor border (CRC, P = 0.010)	Rabbit polyclonal, Sigma 1:100	Lee et al. (2015)
Lee, USA	2016	CRC	49/552, 8.9%	Loss of expression	Suppressor	*ARID1A* loss was associated with mismatch-repair protein deficiency. poor differentiation, lymphovascular invasion, and higher pT stage (all P < 0.05)	Rabbit polyclonal, Sigma, 1:300	Lee et al. (2016)
Agaimy, Germany	2016	Colon, small bowel, and stomach cancer	2/13, 15.4%	Loss of expression	Suppressor	NA	Rabbit polyclonal Abcam, 1:100	Agaimy et al. (2016)
Fountzilas, USA	2018	CRC	16/36, 44%	Loss of expression	Suppressor	*ARID1A* variations independently predicted for unfavorable DFS (HR = 1.99, 95%CI 1.11–3.54, P = 0.020)	NA	Fountzilas et al. (2018)
Wan, China	2018	CRC	3/16, 18.8%	Loss of expression	Suppressor	NA	MygeneSeq technology	Wan et al. (2018)
Sen, USA	2019	CRC	24/164, 14.6%	Loss of expression	Suppressor	The expression of *ARID1A* plays a key role in KRAS-mutated CRC cells	Cell Signaling, 1:500	Sen et al. (2019)
Kishida, Japan	2019	CRC	10/218, 4.6%	Loss of expression	Suppressor	Loss expression of *ARID1A* was significantly correlated to younger age, lymphatic invasion, and lymph node metastasis	Rabbit monoclonal, 1:500	Kishida et al. (2019)
Xu, China	2020	sCRC	1/28, 3.6%	Frameshift variation	Suppressor	*ARID1A* variations and the deficiency of its protein expression were significantly involved in advanced tumor depth, poor differentiation, lymphatic metastasis, BRAF V600E variation, MMR deficiency and MSI phenotype in tumors of CRC patients	NA	Xu et al. (2020)
Tokunaga-1,USA	2020	CRC	468/5726, 8%	Downregulated	Suppressor	*ARID1A* variations could regulate DNA repair pathways	NA	Tokunaga et al. (2020)
Tokunaga-2,USA	2020	CRC	50/619, 8%	Downregulated	Suppressor	*ARID1A* variation was significantly associated with a favourable immune profile indicative of a higher likelihood of response to immune checkpoint inhibitors	NA	Tokunaga et al. (2020)
Tokunaga-3,USA	2020	CRC	104/1099, 10%	Downregulated	Suppressor	*ARID1A* variation was associated with right-sided primary tumor location and earlier tumor stage	NA	Tokunaga et al. (2020)
Tokunaga-4,USA	2020	CRC	58/534, 11%	Downregulated	Suppressor	*ARID1A* variations lead to strong immune activation in CRC	NA	(Tokunaga et al. 2020)
Erfani, Iran	2020	CRC	12/18, 66.7%	Loss or low expression	Suppressor	No significant relationship was found between the loss of *ARID1A* and the OS or the clinicopathological features in CRC	Rabbit antibody Sigma 1:200	Erfani et al. (2020)
Villatoro, USA	2020	Colorectal adenocarcinoma	16/338, 4.7%;	Deficiency	Suppressor	No difference in disease-specific or disease-free survival was found for *ARID1A* deficiency (all *P* > 0.05)	Abcam	Villatoro et al. (2020)
Stein, USA	2020	pCRC PM	pCRC: 179/617, 29%, PM: 42/348, 12%	Variation	Suppressor	NA	Primary antibody clones	Stein et al. (2020)
Wang-1, China	2020	CRC	76/156, 48.7%	Downregulated	Suppressor	NA	NA	Wang et al. (2020)
Wang-2, China	2020	CRC	17/225, 7.6%	Downregulated	Suppressor	NA	NA	Wang et al. (2020)
Jiang, China	2020	CRC	89/1234, 7.2%	Variation	Suppressor	CRC patients with *ARID1A* variation showed a significantly longer DFS/PFS (HR = 0.74, *P* = 0.0026)	NA	Jiang et al. (2020)
Huang, China	2021	CRC	65/630, 10.3%	Variation	Suppressor	NA	NA	(Huang et al. 2021)
Perna, Spain	2021	HG-CRCs	12/29, 41.4%	Loss of expression	Suppressor	The differences in survival were not statistically significant (HR = 0.58, 95% CI = 0.23–1.49, P = 0.257)	Polyclonal Sigma, 1:500	Perna et al. (2021)
Kamori, Japan	2021	CRC	20/201, 10%	Variation	Suppressor	Tumor histological grade was significantly correlated with *ARID1A* variation status in those patients with right-sided CRC	Rabbit polyclonal,	Kamori et al. (2021)

*ARID1A* AT-rich interactive domain 1A, *CRC* colorectal cancer, *HR* Hazard ratio, *OR* odds ratio, *OS* overall survival, *DFS* disease-free survival, *HG-CRC* high grade colorectal carcinomas, *RCC* right-sided colorectal cancer, *LCC* left-sided colorectal cancer, *pCRC* primary colorectal cancer, *NA* not available, *PFS* progression-free survival, *RFS* recurrence-free survival

Since the variation rate, clinical significance, and biological function of *ARID1A* are different in 23 qualified studies, we have therefore conducted an in-depth review of these eligible studies as follows.

### ARID1A expression and variations in CRC

#### Variation rate of ARID1A in CRC among different studies

Comprehensive genome analysis is one useful tool to identify variations of various oncogenes and tumor-suppressor genes, particularly in those genes that code for chromatin remodeling factors (Centore et al. 2020; Goswami et al. 2020; Mao et al. 2013; Mathur 2018; Wei et al. 2014; Ye et al. 2014) One of such genes is *ARID1A.* However, the variation rate of *ARID1A* in CRC is low, Jones et al. (Jones et al. 2012) reported 10%, and Kim et al. (49) did not find variations. Based on this evidence, the effect of *ARID1A* loss in CRC is still underestimated. Wei et al. (2014) found that the *ARID1A* protein loss caused by immunohistochemistry occurred in 25.8% of primary CRC tumors, and the proportion was higher in stage IV CRC, which was 35.2%, suggesting that *ARID1A* protein loss is not very common in CRC.

In this narrative review, the evidence related to the *ARID1A* variants in CRC was comprehensively summarized. According to the data of 23 eligible studies, great different has been identified on the *ARID1A* variation rate varies greatly. As shown in Fig. 2, *ARID1A* variation in pCRC, PM-CRC, and HG-CRC was recorded at 29%, 12%, and 41.4%, respectively. Based on the previous publications, the highest variation rate of *ARID1A* in ovarian clear cell carcinoma, which is as high as 46–57% (Yoshino et al. 2020). But CRC did not make any comments or conclusions.

CRCs have two genetic and clinically distinct subtypes: chromosomal instability tumors (CIN) and microsatellite instability tumors (MSI). Most tumors show CIN, and about 15% are MSI. In MSI tumors, the mismatch repair (MMR) system is defective, which can usually correct a large number of errors that occur during DNA replication. This leads to a large number of small insertions and deletions in the repetitive regions surrounding the genome, especially in the short tandem repeat regions called microsatellites. The overall variation rate of MSI-CRC is estimated to be about 10 times that of microsatellite stable (MSS)-CRC (2012; Vogelstein et al. 2013). Tokunaga et al. (2020) compared the relationship between *ARID1A* variations and the molecular characteristics in CRC by using the next-generation sequencing, RNA sequencing, and the immunohistochemistry methods. They found that *ARID1A* variations were more common in primary and early age tumors on the right site of CRC. *ARID1A* mutant tumors mainly have gene variations related to chromatin modification, DNA repair, WNT signaling pathway, and EGFR inhibitor resistance pathway at the same time, and *ARID1A* variations have a strong regulatory effect on DNA repair pathways. CMS1, one of the consensus molecular subtypes of the CRC classification system, plays an essential role in immune response (Guinney et al. 2015). It was reported that *ARID1A* mutant samples proved a higher prevalence of CMS1 than *ARID1A* wild-type samples, which indicates *ARID1A* variation could result in strong immune activation (Tokunaga et al. 2020).

The data from the 23 eligible studies showed that the *ARID1A* variation rates and expression levels were different among different studies. These discrepancies might be related to various factors, e.g., different demographic features (i.e., sample size and race), CRC stage (early or advanced), different antibodies of anti-*ARID1A*, the assessments of the *ARID1A* protein expression (i.e., IHC, western blot, targeted sequencing analysis, qRT-PCR, tissue microarrays, and chromatin immunoprecipitation), and multifarious co-present or targeted genes being affected.

All studies indicate that *ARID1A* is low or absent in CRC, and *ARID1A* acts as a tumor suppressor, which is consistent with the function of *ARID1A* in other types of cancer (Wu and Roberts 2013). According to the current evidence, *ARID1A* variants are only expected to play a tumor suppressor effect CRC development.

#### Clinical significance of ARID1A in CRC

Although the high frequency of *ARID1A* variants has been observed in CRC, the prognostic value of *ARID1A* in CRC is still controversial. Jiang et al. (2020) found that DFS or PFS of patients with variations in *ARID1A* was significantly prolonged (HR = 0.74, 95%CI: 0.64–0.91, P = 0.0026). The OS of patients with *ARID1A* variation was significantly prolonged than those with wild-type (28 months vs. 18 months, *P* = 0.0092). In other words, *ARID1A* deletion predicts superior OS in stage IV CRC. Wei et al. (2014) analyzed 209 primary CRC tumor samples by IHC and discovered that *ARID1A* loss was detected in fifty-four (25.8%) primary CRC tumors. Moreover, the authors also observed that the distant metastasis rate was higher in patients with *ARID1A* loss than those without *ARID1A* loss (46.3% vs. 29.7%). In addition, Wei et al. further observed that *ARID1A* loss was related to the late TNM stage (*P* = 0.020) and poor pathological classification (*P* = 0.035). However, this study highlighted that positive *ARID1A* was associated with worse OS as compared to those with negative *ARID1A* in stage IV CRC (HR = 2.49, 95% CI: 1.13–5.51), indicating that *ARID1A* loss predicted superior OS in stage IV CRC. Largely consistent with Wei et al.’s findings, (Ye et al. 2014) found that *ARID1A* was related to tumor staging, lymphatic invasion, and tumor recurrence of CRC. *ARID1A*-deficient CRC has a higher proportion of lymph node and distant metastasis, and the overall 5-year survival rate shows a downward trend. Kishida et al. (2019) proved that lymphatic invasion is independently related to *ARID1A* expression. The above studies confirmed that the prognostic value of *ARID1A* variants in CRC is related to *ARID1A* defect or low expression. Tokunaga et al. (2020) believed that the *ARID1A* variation was related to the location of the primary tumor on the right side and the stage of the early tumor. The above data suggest that both low and high expression of *ARID1A* variants are related to the prognostic significance of CRC.

However, other studies did not support a positive association between the *ARID1A* variant or expression level and the prognosis of the disease in the CRC. Chou et al. (2014) found that there is a strong correlation between *ARID1A* expression loss and older age, right-sided tumors, larger tumor size, medullary morphology, high histological grade, BRAFV600E variation, and loss of mismatch repair protein expression (all *P* < 0.01), however, no significant association was found between loss of *ARID1A* expression and overall survival. Similarly, Lee et al. (2015) have also found that the loss of *ARID1A* expression was significantly related to the negative lymphatic invasion of CRC (*P* = 0.003), and tumor boundary expansion (CRC, *P* = 0.010). But there is no obvious correlation between *ARID1A* expression and 5-year OS. Lee et al. (2016) suggested that at a median follow-up of 49 months, *ARID1A* deletion was not associated with the OS, disease-specific survival, or recurrence-free survival in CRC patients.

Based on these studies, there is no significant correlation between *ARID1A* variants and the survival of CRC. One potential explanation for this observation may be due to the low number of CRC cases in some studies. For instance, the study by Erfani et al. (2020) reported that the *ARID1A* variation rate in CRC was as high as 66.7%. The authors found that among the 18 CRC tumors studied, 7 cases (38.8%) and 5 cases (27.7%) had no or low ARID1A expression, respectively. The limited number of patients may limit the study’s results. Conversely, studies involving a relative large number of patients are more likely to determine the poor prognostic significance of *ARID1A* variants in CRC (Fountzilas et al. 2018; Jiang et al. 2020; Xie et al. 2014). Certainly, various anti-*ARID1A* antibodies being used in every study conducted by IHC (e.g., antibody’s clone, manufacturer, dilution rate, IHC score, and cut-off value). These factors may be the underlying reasons behind the different results obtained in each study.

In summary, *ARID1A* variants may be predictive of metastasis, recurrence, and death of CRC patients, which indicates that *ARID1A* may play a crucial role in the development of CRC. It is worth noting that because some studies do not support the prognostic value of *ARID1A*, further studies are needed to verify the prognostic significance of *ARID1A* variants in CRC.

#### Molecular mechanisms of ARID1A variations on CRC

Since the causal association between *ARID1A* variation and CRC has been observed in multiple clinical studies, an exhaustive comprehension of the molecular functions of *ARID1A* is of great significance to researchers. *ARID1A* is a driver gene that encodes the DNA binding subunit of the SWI/SNF chromatin-remodeling complex. *ARID1A* provides specificity for the SWI/SNF complex and promotes protein–protein or protein-DNA molecular interactions. *ARID1A* inactivation may activate the cell cycle process, resulting in uncontrolled cell proliferation of cancer cells, indicating that *ARID1A* is a potential tumor suppressor function and the correlation between *ARID1A* deletion and tumorigenesis (Nagl et al. 2005). *ARID1A* might exert its biological functions and pathological impact on CRC by interacting with multiple mutated genes, affected signaling pathways, and some other factors.

#### ARID1A variation was associated with the co-occurrence variation of TP53 and some other genes

Some authors believe that there is a link between *ARID1A* and TP53 variations. TP53 (also named P53) is one of the most common genetic variants in human cancers and plays an important role in the regulation of the apoptosis, cell cycle, and DNA repair (Pinto et al. 2020). The variation of TP53 has become a critical biomarker of cancer prognosis due to its cancerous biological function. Guan et al. (2011) proposed the theory that *ARID1A* and p53 inhibit tumor growth synergistically at the molecular level. Other researchers suggested that *ARID1A* and TP53 variations are reciprocally exclusive and in charge of alternative pathways of tumorigenesis (Jones et al. 2012; Wang et al. 2011). In gastric cancer and gynecological cancer, *ARID1A* variation or loss of *ARID1A* protein expression is closely related to microsatellite instability, and negatively related to the variation of TP53 (Bosse et al. 2013). Tokunaga et al. (2020) reported that among the 20 genes assessed in the CRC cohort, only TP53 variations and *ARID1A* variations were reciprocally exclusive. *ARID1A* variation cause defects in cell cycle control point activation and TP53 variation in answer to DNA damage (Watanabe et al. 2014). *ARID1A* and TP53 jointly prevent tumorigenesis by inhibiting the transcriptional activation of genes downstream of tumors. As a result, the prognostic significance and biological effects of *ARID1A* in CRC may partly depend on the variation of TP53.

TP53 and *ARID1A* are considered to be the most common mutant genes in CRC (Stein et al. 2020). In addition to TP53, *ARID1A* variations can also occur simultaneously and may interact with some other genes (such as APC, FBXW7, PIK3CA, PD-L1, and KRAS), which may be involved in the development of CRC. Numerous studies have shown that *ARID1A* variations are often accompanied by Adenomatous polyposis coli (APC) variations in CRC. It was reported that the APC tumor suppressor is mutated in 27–71.7% of the CRC cases (Ashktorab et al. 2019; Huang et al. 2021). *ARID1A* and APC variations could increase the proliferation and survival of the CRC cells (Sen et al. 2019). It was reported that FBXW7 was one of the most frequently mutated genes of Chinese CRC patients (Liu et al. 2018). *ARID1A* variations are frequently accompanied by FBXW7 variations. Huang et al. (2021) found that both FBXW7 (17.5%) and *ARID1A* (10.3%) were the most common mutated genes in CRC patients via a genomic alteration analysis. Wang et al. (2020) showed that *ARID1A* (7.6%) and FBXW7 (6.2%) frequently mutated in the deficient mismatch repair CRC. In a study of the African Americans population, Ashktorab et al. (2019) demonstrated that *ARID1A* (7%) and FBXW7 (4%) were the common variants in CRC patients. PIK3CA is an oncogene in CRC. A comparative genomic analysis demonstrated that variations in *ARID1A* and PIK3CA (6.7%) genes between primary CRC and metastatic liver tumors of CRC (Lee et al. 2014). A genes exome sequencing study (Ashktorab et al. 2019) reported that the variation rate of *ARID1A* is 7% (8/121), while in PIK3CA is 6% (7/121) in CRC, and both two genes contributed to the carcinogenic process of CRC.

The programmed death-1 (PD-1)/programmed death-ligand 1 (PD-L1) axis is one of the effective therapeutic targets for immune checkpoint blockade therapy. Kamori et al. (2021) reported that CRC with *ARID1A* variations was likely to have a higher tumor mutational burden, while *ARID1A*-deficient CRC was frequently accompanied by enhanced PD-L1 expression by stromal cells. Kirsten rat sarcoma viral oncogene homolog (KRAS) and *ARID1A* variants have also been found by many researchers to coexist in CRC development. Several activation-type KRAS variations are observed in the group positive for the protein expression of *ARID1A*. The existence of *ARID1A* variations (44%) and KRAS variations (48%) has been demonstrated in stage I–III CRC (Fountzilas et al. 2018). Sen et al. (2019) suggested that *ARID1A* might facilitate KRAS signaling-regulated enhancer activity in CRC. They found that KRAS variations were particularly dependent on the presence of *ARID1A.* According to several reports, along with *ARID1A* variations*,* the KRAS variations rate were recorded ranging from 4.3% to 50% (Ashktorab et al. 2019; Cajuso et al. 2014; Huang et al. 2021). In KRAS mutant cells, after *ARID1A* is deleted, the enhancer co-occupied by *ARID1A* and AP1 transcription factors become inactive, resulting in a decrease in target gene expression (Sen et al. 2019). Therefore, in CRC with KRAS variation, mSWI/SNF complex may provide a unique and context-dependent treatment option.

#### Roles of the ARID domain-containing gene family

The ARID domain-containing gene family might also contribute to the tumorigenesis mechanisms of CRC, and act collectively with *ARID1A* variations. It was described *ARID1A* as belonging to the ARID domain-containing gene family (Cajuso et al. 2014). ARID1B (13%, 6/46), ARID2 (13%, 6/46), ARID4A (20%, 9/46) and *ARID1A* (39%, 18/46) was reported to frequently have variations in tumors. The results show that besides *ARID1A*, other members of the ARID gene family might also play a part in MSI CRC. Jones et al. (2012) evaluated 759 malignant tumors, including pancreas, breast, colon, stomach, lung, prostate, brain, and blood (leukemia). And truncated variations were found in 6% of the tumors studied; non-truncated cell variations were found in another 0.4% of tumors. Variations are most common in gastrointestinal samples, and 12 of 119 (10%) colon samples have *ARID1A* variations. The majority of the mutant colorectal tumors show microsatellite instability (MSI). The variations in these tumors are insertions or deletions of single nucleotide repeats outside the frame.

#### Roles of the affected signaling pathways

The pro-oncogenic roles of *ARID1A* variation on CRC development may also associate with its regulation on the activity of several affected signaling pathways. Some investigators even believe that variations in some tumor pathways are involved in the first step of progress from normal to CRC (Suleiman et al. 2015). Crosstalk between *ARID1A* and PI3K/Akt pathway has been detected in multiple cancers (Sun et al. 2021). Xie et al. (2014) believed that *ARID1A* depletion could promote CRC cell proliferation, enhance chemoresistance, and inhibit cell apoptosis by regulating the activity of the Akt signaling pathway. MTT experiments showed that overexpression of *ARID1A* in SW620 cells led to decreased cell proliferation, and depletion of *ARID1A* could increase cell growth rate. Sen et al. (2019) found that *ARID1A* has a previously unknown background-dependent tumor support function in CRC downstream of the KRAS signal and MEK/ERK pathway, showing that the absence of *ARID1A* enhances the proliferation of CRC cells. In addition, at the transcriptional level, the authors also detected a strong colocalization of *ARID1A* and TCF7L2, a downstream effector of the Wnt pathway. Aurora kinase A (AURKA) commonly functions in mitosis and non-mitotic biological processes. Wu et al. (Wu et al. 2018) demonstrated that *ARID1A* loss contributed to the growth and survival of the CRC cells via negatively regulating AURKA-mediated signaling and the downstream genes, such as PLK1 and CDC25C. A gene set enrichment analysis conducted by Tokunaga et al. showed that *ARID1A* mutant status was closely correlated to the DNA repair pathway, mediating chemotherapy/radiotherapy sensitivity of CRC (Tokunaga et al. 2020). Since intestinal deletion of *ARID1A* was tightly associated with CRC development, Hiramatsu et al. believed the underlying molecular mechanisms might be related to the disruption of the intestinal homeostasis, and pointed out that the Wnt signaling pathway crucially involved this action.

#### ARID1A variation associated with MMR deficiency and hypermethylation

MMR deficiency is one of the important prognostic factors in CRC. The significant association between *ARID1A* deletion and MMR defect in CRC has been fully demonstrated in the literature, showing that loss *ARID1A* expression in 15–25% of MMR-deficient versus 4–6% of MMR-intact CRC cases, respectively (Agaimy et al. 2016). Lee et al. (Lee et al. 2016) reported that *ARID1A* loss was significantly more prevalent in the MMR-deficient CRC cases than in the MMR-proficient CRC cases (18.7% *vs* 6.3%, *P* < 0.001). A previous study (Ye et al. 2014) indicated that *ARID1A* variations were associated with a worse outcome among the MMR-abnormal CRC cases. This study also demonstrated that the main mechanism of MMR deficiency in *ARID1A*-deficient tumors was hypermethylation of the mutL homolog 1 (MLH1) gene promoter (Ye et al. 2014). BRAF V600E variations are frequently shown in these MMR-deficient tumors with *ARID1A* deletion. By comparison, MMR defects due to germline variations (i.e., Lynch syndrome) appear to occur mainly in *ARID1A*-preserving cases (Chou et al. 2014; Ye et al. 2014). The association between *ARID1A* deletion and MMR defect co-exists in the early CRC. In addition, most of these MMR-deficient *ARID1A* deletion tumors do show simultaneous deletion of MLH1 and PMS2, and this pattern is expected in tumors where the MLH1 promoter is methylated (Lee et al. 2016). Chou et al. (Chou et al. 2014) believe that, considering these associations, *ARID1A* may be used as a marker of somatic hypermethylation for the classification genetic testing of Lynch syndrome. It is worth noting that the MMR defect pattern that suggests Lynch syndrome can also occur in tumors with *ARID1A* deletion. Promoter hypermethylation is one of the main reasons for *ARID1A* variations. *ARID1A* loss leads to epigenetic alterations by a deficient SWI/SNF complex with subsequent MLH1 promoter methylation. Chou et al. reported that a low level of *ARID1A* was closely associated with larger tumor size, right-sided tumors, and high histological grade of CRC, which were features of somatic hypermethylation (Chou et al. 2014). Erfani et al. (2020) found that promoter DNA hypermethylation significantly promoted the silencing or down-regulation of *ARID1A* in CRC cell lines. The authors also suggested that *ARID1A* might be an effective tumor suppressor gene in certain subtypes of CRCs because it affects many genes through its role in chromatin remodeling expression (Erfani et al. 2020). Based on the above evidence, promoter hypermethylation may serve as a down-regulation mechanism of *ARID1A* in CRC.

**Fig. 3:**
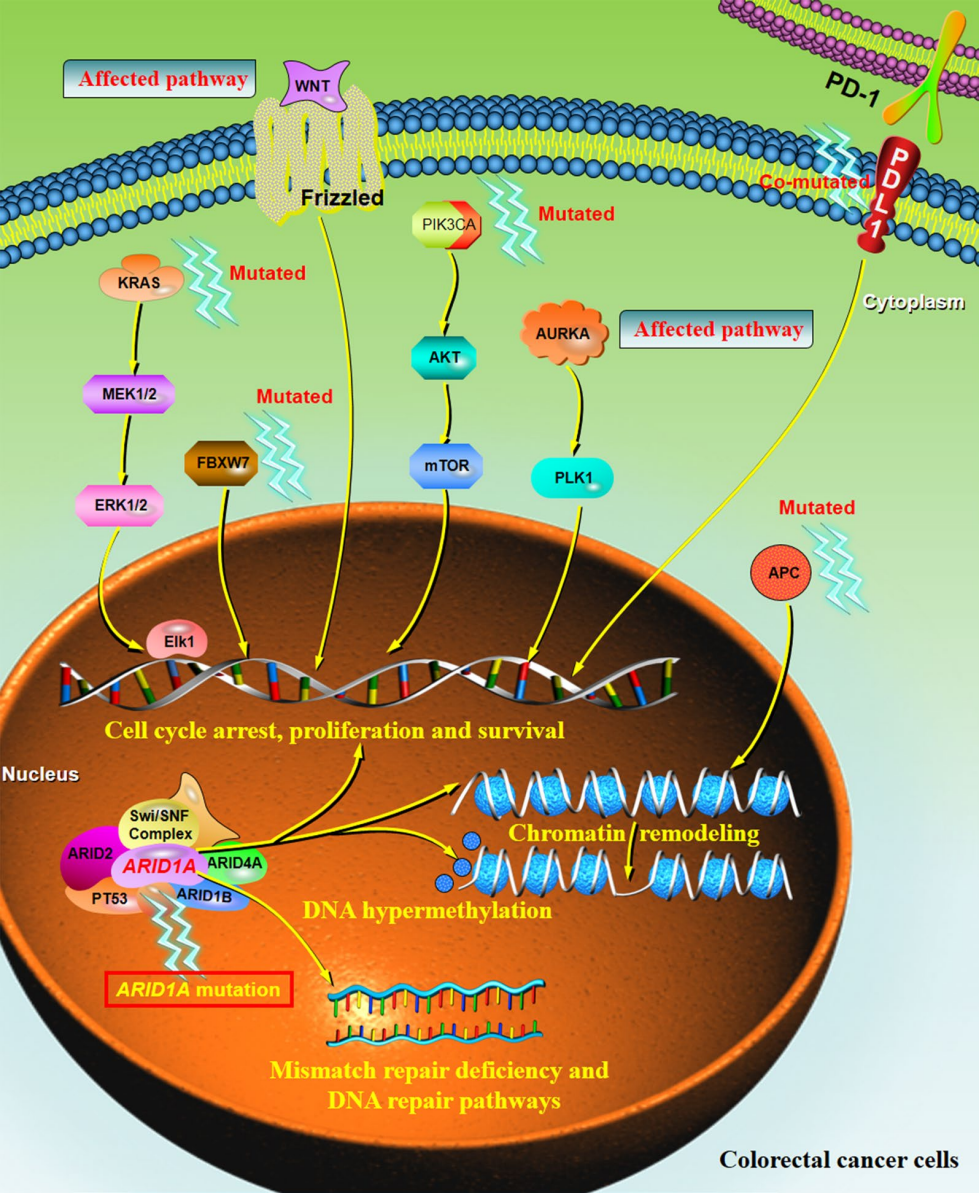
The mechanism by which the *ARID1A* variation contributes to the pathogenesis of CRCs. *ARID1A,* a subunit of the chromatin remodeling protein SWI/SNF, is considered to be associated with the tumorigenesis and the progression of CRCs. The process is initiated by the mutation of multiple genes (i.e., TP53, ARID domain-containing gene family, APC, FBXW7, PIK3CA, PD-L1, and KRAS), the dysregulation of several signaling pathways (i.e., PI3K/Akt signaling, MEK/ERK pathway, WNT pathway, AURKA-mediated signaling, and DNA repair pathways), chromatin remodeling, mismatch repair deficiency, and DNA hypermethylation, leading to the cell cycle arrest, proliferation, and survival of the CRC cells. *ARID1A* AT-rich interaction domain 1A, *APC* adenomatous polyposis coli, *FBXW7* F-Box and WD repeat domain containing 7, *PIK3CA* phosphatidylinositol-4,5-bisphosphate 3-kinase catalytic subunit α, *SWI/SNF* SWItch/Sucrose non-fermenting, *PD-L1* programmed death ligand 1, *KRAS* Kirsten rat sarcoma viral oncogene homolog, *AURKA* aurora kinase A

In summary, *ARID1A* variants seem to play an important role in the occurrence and progression of CRC tumors. As illustrated in Fig. 3, this schematic diagram summarizes the multi-factor mechanisms that may be involved in the development of *ARID1A*-driven CRC, including cell cycle arrest, chromatin remodeling and chromosome organization, and DNA hypermethylation. The interactions of multiple genes (i.e., TP53, APC, FBXW7, PIK3CA, PD-L1, and KRAS) and the affected signaling pathways (i.e., PI3K/Akt, MEK/ERK pathway, Wnt pathway, AURKA-mediated signaling, and DNA repair pathway) enhance the process of cell proliferation and anti-apoptosis. Nevertheless, further relevant studies are still needed to better clarify the potential mechanism of *ARID1A* variations that trigger the development of CRC.

### Limitations and perspectives

This is the first study to comprehensively review *ARID1A* variations associated specifically with CRC from the clinical through the molecular level. However, several drawbacks in the present study should be acknowledged. First, though *ARID1A* variation is closely associated with the clinicopathologic features of CRC (i.e., TNM stage, tumor location, and histological grade), its role on the prognostic significance of CRC remains controversial among the 23 eligible studies, especially on the survival. Second, large differences in the variation rate of *ARID1A* in CRC were observed among different included studies, ranging between 3.6 and 66.7%. This heterogeneity might be partly due to various geographic populations, study design, sample size, different tumor staging, gender, age, and the assessments for the expression level of *ARID1A*. Third, the biomarker role, the potential antitumor effect, and the underlying biological mechanisms for the participation of *ARID1A* variants in the tumorigenesis of CRC, development, prediction, and therapy need to be further studied.

## Conclusions

In the present review, all of the 23 included studies consistently suggest that *ARID1A* is a tumor suppressor in CRC. The loss of *ARID1A* expression may represent the *ARID1A*-driven carcinogenesis in CRC. However, the rate of *ARID1A* variation in CRC cases is diverse across different studies, ranging from 3.6 to 66.7%. Though *ARID1A* variation status has several clinical impacts on CRC, such as serving as a biomarker for survival prognosis and various therapies, no significant differences were observed between the variation and wild type of *ARID1A* in a few studies. The biological functions and pathological impacts of *ARID1A* variations on CRC might be correlated to the co-occurrence variation of other genes (i.e., TP53 and KRAS) and the regulation of signaling pathways (i.e., Akt signaling and WNT signaling). Upon further validation with the clinical and biological features of *ARID1A* variations in CRC by future studies, *ARID1A* has the potential to serve as an important prognostic factor and individualized therapeutic target for CRC.

## Data Availability

Not applicable.

## Abbreviations

CRC: Colorectal cancer
*ARID1A*: AT-rich interaction domain 1A
*SWI/SNF*: SWItch/sucrose non-fermenting
APC: Adenomatous polyposis coli
FBXW7: F-box and WD repeat domain containing 7
PIK3CA: Phosphatidylinositol-4,5-bisphosphate 3-kinase catalytic subunit α
PD-L1: Programmed death ligand 1
KRAS: Kirsten rat sarcoma viral oncogene homolog
AURKA: Aurora kinase A
